# El Niño Southern Oscillation as an early warning tool for dengue outbreak in India

**DOI:** 10.1186/s12889-020-09609-1

**Published:** 2020-10-02

**Authors:** Malay Pramanik, Poonam Singh, Gaurav Kumar, V. P. Ojha, Ramesh C. Dhiman

**Affiliations:** ICMR-National Institute of Malaria Research, New Delhi, 110077 India

**Keywords:** ENSO, Dengue case index, Early warning, Indian Ocean dipole, Monsoon, Post monsoon

## Abstract

**Background:**

Dengue is rapidly expanding climate-sensitive mosquito-borne disease worldwide. Outbreaks of dengue occur in various parts of India as well but there is no tool to provide early warning. The current study was, therefore, undertaken to find out the link between El Niño, precipitation, and dengue cases, which could help in early preparedness for control of dengue.

**Methods:**

Data on Oceanic Niño Index (ONI) was extracted from CPC-IRI (USA) while the data on monthly rainfall was procured from India Meteorological Department. Data on annual dengue cases was taken from the website of National Vector Borne Disease Control Programme (NVBDCP). Correlation analysis was used to analyse the relationship between seasonal positive ONI, rainfall index and dengue case index based on past 20 years’ state-level data. The dengue case index representing ‘relative deviation from mean’ was correlated to the 3 months average ONI. The computed r values of dengue case index and positive ONI were further interpreted using generated spatial correlation map. The short-term prediction of dengue probability map has been prepared based on phase-wise (El Niño, La Niña, and Neutral) 20 years averaged ONI.

**Results:**

A high correlation between positive ONI and dengue incidence was found, particularly in the states of Arunachal Pradesh, Chhattisgarh, Haryana, Uttarakhand, Andaman and Nicobar Islands, Delhi, Daman and Diu. The states like Assam, Himachal Pradesh, Meghalaya, Manipur, Mizoram, Jammu & Kashmir, Uttar Pradesh, Orissa, and Andhra Pradesh shown negative correlation between summer El Niño and dengue incidence. Two - three month lag was found between monthly ‘rainfall index’ and dengue cases at local-scale analysis.

**Conclusion:**

The generated map signifies the spatial correlation between positive ONI and dengue case index, indicating positive correlation in the central part, while negative correlation in some coastal, northern, and north-eastern part of India. The findings offer a tool for early preparedness for undertaking intervention measures against dengue by the national programme at state level. For further improvement of results, study at micro-scale district level for finding month-wise association with Indian Ocean Dipole and local weather variables is desired for better explanation of dengue outbreaks in the states with ‘no association’.

## Background

Dengue is an emerging vector borne disease due to rapid urbanization, scarcity of water and changing climatic conditions all over the globe. The transmission of dengue has close interaction of vector, virus and human host, of which Aedes vectors are highly sensitive to climatic and environmental factors [1]. Owing to inter-annual variability in climatic conditions, the sudden occurrence of dengue (outbreaks) is generally witnessed in many subtropical and tropical countries of the world [2, 3]. As per World Health Organization [4], dengue is a rapidly growing mosquito-borne arbo-viral disease in the world which has increased 30-fold in the last 50 years. The geographic distribution of this disease is extending its spatial and temporal limits to even new countries and from urban to rural areas [4]. An estimated 390-million dengue infections occur annually [5], and 2.5 billion population live in dengue-endemic countries [6] posing a serious public health challenge [7].

Several studies reveal that the dengue outbreaks are characterised by seasonal and multi-annual pattern of occurrence [8]. Studies undertaken in several parts of the world provide evidence that the periodic outbreaks of dengue are closely associated with local weather conditions and climate cycle of El Niño Southern Oscillation (ENSO) [9–11], but the risk of outbreaks differ based on the strength of ENSO [12, 13]. The ENSO is a naturally occurring, large scale, inter-annual climatic phenomenon worldwide which occur at 2–7 years’ interval over the tropical Pacific and Indian Ocean region [14, 15]. The Southern Oscillation is the accompanying atmospheric component, coupled with the sea temperature change: warm phase (El Niño) is accompanied by high air surface pressure in the tropical western Pacific and cold phase (La Niña) with low air surface pressure which trigger a significant number of climatic anomalies [14]. The cycle of cooling (La-Nina) and warming (El Nino) of sea surface temperatures (SST) in the Eastern Equatorial Pacific (Darwin, Australia and Tahiti) resulting from the changes in the oceanic circulation and closely interlinked to changing the air pressure in the East and West Pacific (Southern Oscillation) [15]. Therefore, ENSO influences the regional weather pattern (i.e., temperature and rainfall) in certain geographic regions of the world [2]. In India, El-Niño has been generally known to suppress monsoon rainfall while La-Nina increases it [16–18]. Therefore, fluctuations in ENSO are likely to be associated with inter-annual variation in occurrence of dengue and could even trigger the outbreaks. In a recent study, weak synchronous correlation has been found between Niño 3.4, dipole mode index (DMI; represented by anomalous sea surface temperature gradient between the western equatorial Indian Ocean), and dengue cases of 2010–2017 based on over dispersed datasets [19].

**Fig. 1:**
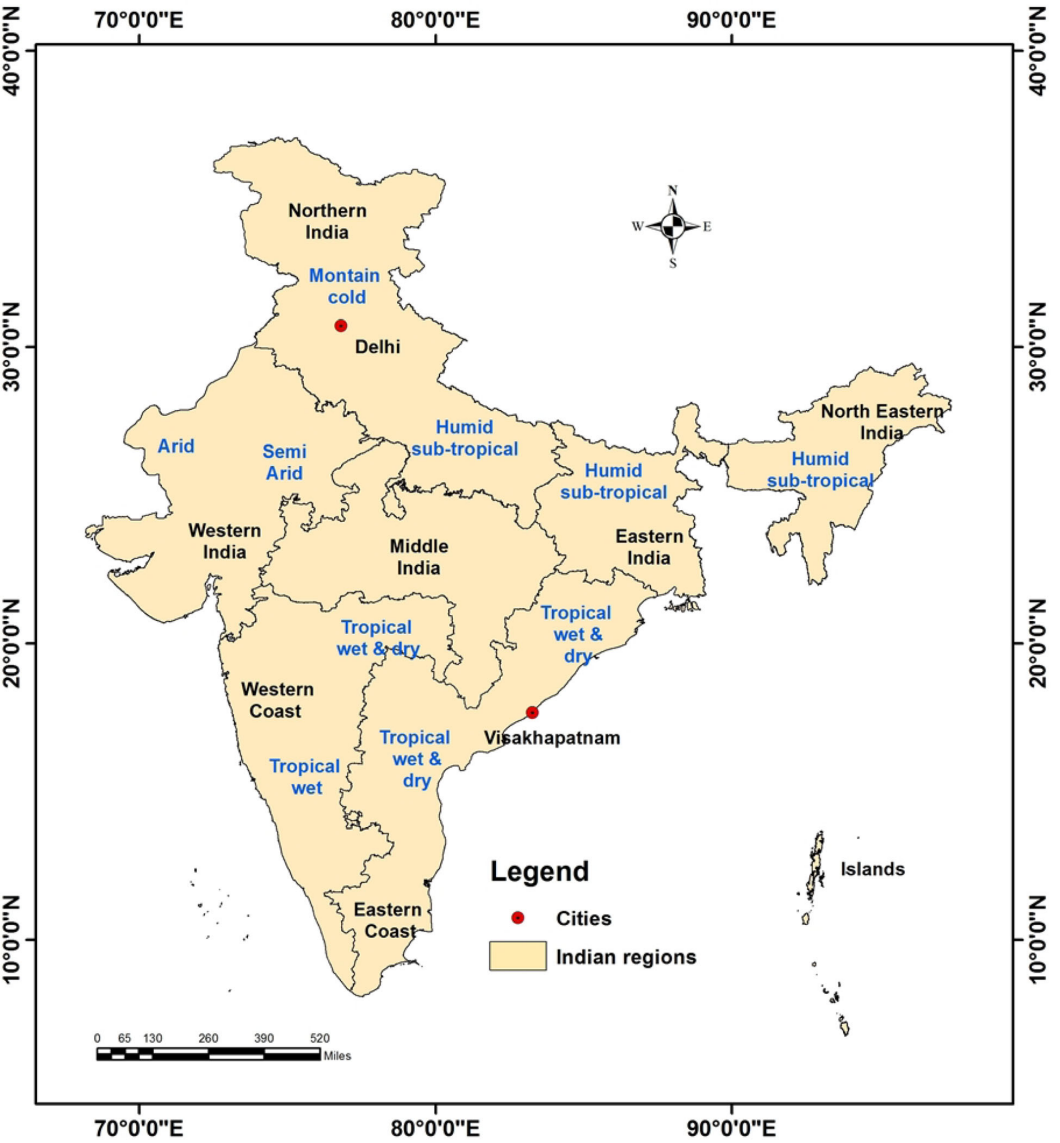
Broad geographical regions with seasonal climatic characteristics in India. Red points show the cities location selected for the local-scale analysis. The shapefiles used for the map were open access provided by ESRI ArcGIS Online (2018). The map was generated using ESRI ArcMap 10.2

India is a federal union comprising of 28 states and 8 union territories, with large variation in geography and area (Fig. 1). It has very diverse climatic conditions with four different climatic seasons, where July–September months are considered as monsoon period and October–December is post-monsoon period [20]. In this period, over 80% of total annual rainfall is received by the country [20]. In contrast, during the post-monsoon months (October to December), a different monsoon cycle, the northeast (or “retreating”) monsoon, brings dry, cool, and dense air masses to large parts of India [20]. Most of the dengue cases occur during the monsoon (July to September) and post-monsoon (October to December) season [19, 21]. Therefore, the association between dengue outbreaks, pre and post-monsoon precipitation, and ENSO at least at the state level is necessary. The present work focuses on finding the relationship between ENSO, rainfall and dengue cases in the post-monsoon and monsoon season in India. The generated maps can help understand the seasonal variability of dengue outbreaks and would help in timely response/preparedness by the National Vector Borne Disease Control Programme (NVBDCP) towards outbreaks.

## Methods

### Data collection

Annual state-wise dengue cases datasets were collected from the ‘National Health Profile Reports’ for the year of 1994–2009 (Central Bureau of Health Intelligence (CBHI) http://cbhidghs.nic.in/ [accessed on 2 Apr 2018] and the website of NVBDCP for the year 2010–2017 (www.nvbdcp.gov.in/ accessed on Apr 2, 2018) [22]. Monthly cases of dengue in respect of Delhi (1996–2016) and Vishakhapatnam (2014–2017) were procured from the office of Medical Health Officers, Municipal Corporation of Delhi and State Programme Officer, Andhra Pradesh respectively. State-wise monthly rainfall data from 1951 to 2016 were collected from annual summary reports of India Meteorological Department (IMD) (http://www.imdpune.gov.in/ accessed on Feb 1, 2018) [23]. The three-month running mean of ONI in the Niño 3.4 regions (longitude 120°W-170°W and latitude 5°N-5°S) were collected from Climate Prediction Center-National Oceanic and Atmospheric Administration (CPC-NOAA) (http://www.cpc.ncep.noaa.gov/products/analysis_monitoring/ensostuff/ensoyears.shtml visited on Mar 4, 2018) [24]. The predicted data of ENSO were extracted from the IRI, Columbia University for the year of 2019. The ONI values of + 0.5 or higher (positive ONI) were considered as El Niño conditions, + 0.5 to − 0.5 values as Neutral, and --0.5 or lower values (negative ONI) were considered as a La Niña condition [24].

### Data processing

As the spatio-temporal fluctuation in dengue cases is very high in tropical countries like India, the cases were brought to a single measure unit. The reported cases were converted to 3 months averaged dengue case index. The ‘dengue case index’ (eq. 1) calculated by ‘the relative deviation of the number of dengue cases from mean cases’, is expressed as

1 $$ \mathrm{Case}\ \mathrm{index}=\frac{\mathrm{x}-\dot{\mathrm{x}}}{\dot{\mathrm{x}}} $$

Where x represents average dengue cases, and $\dot{\mathrm{x}}$ indicates the mean annual dengue cases in the Indian states.

Similarly, rainfall index (eq. 2) was calculated by the ‘percent deviation rainfall from mean’.

2 $$ \mathrm{Rainfall}\ \mathrm{index}=\frac{\mathrm{x}-\upmu}{\upmu}\times 100 $$

Where x implies a seasonal average in a year and μ implies mean seasonal rainfall over the period of study year (1951–2016). As the present study considered the dengue cases analysis for monsoon and post-monsoon period, therefore, July–September and October–December months were considered for the index, respectively.

### Data analysis and presentation

As the data is normally distributed based on the Shapiro-Wilk test, at 5% significant level, the association between positive ONI and rainfall index, dengue case index and ‘rainfall index’, and positive ONI and dengue case index was evaluated using product-moment correlation coefficient (r). The ‘r’ value of ≥0.5 and ≤ − 0.5 was selected for positive and negative correlation, respectively, at 5% significant level (P). The season-wise computed R-values between ‘dengue case index’ and positive ONI values were imported to Environmental Systems Research Institute (ESRI) Arc-GIS platform to create spatial correlation map. The algorithm ‘Inverse distance weighted’ (IDW) was used to spatially interpolate all the correlated values, creating a more precise and smoother surface from sparsely distributed datasets.

### Delineation of different states based on ENSO outlook of 2020

To understand the phase-wise relationship between ENSO and dengue, historical Oceanic Niño Index values were first divided into different phases, (i.e., El Niño, Neutral, and La Niña) and then correlated with dengue cases. The average ONI values of April, May, and June were considered as positive summer ONI, and the average ONI values of July, August, and September were considered as positive monsoon ONI. Thereafter, short-term probabilities of dengue outbreaks were mapped on the basis of the predicted phase of ENSO in 2020.

### Local-scale analysis

Delhi city was selected for the local scale analysis as an example of land-locked city where monsoon rainfall is moderate (700–750 mm) as compared to other parts of India. Visakhapatnam city was selected as an example of coastal city, where yearly average rainfall is moderate to high (1400–1600 mm) (Fig. 1). Moreover, in these cities’ dengue outbreaks are more frequent. Data on the incidence of dengue were collected from the Municipal Corporation, Delhi (MCD) and the State Programme Officer, Andhra Pradesh for Delhi and Visakhapatnam cities respectively. Pearson’s correlation test was adopted to find out the lag months’ association between positive ONI values and dengue cases; and rainfall and dengue cases based on 21 years (1996–2016) monthly data of Delhi and 4 years (2014–2017) data of Visakhapatnam city.

## Results

### Correlation between positive ONI and rainfall index

**Fig. 2:**
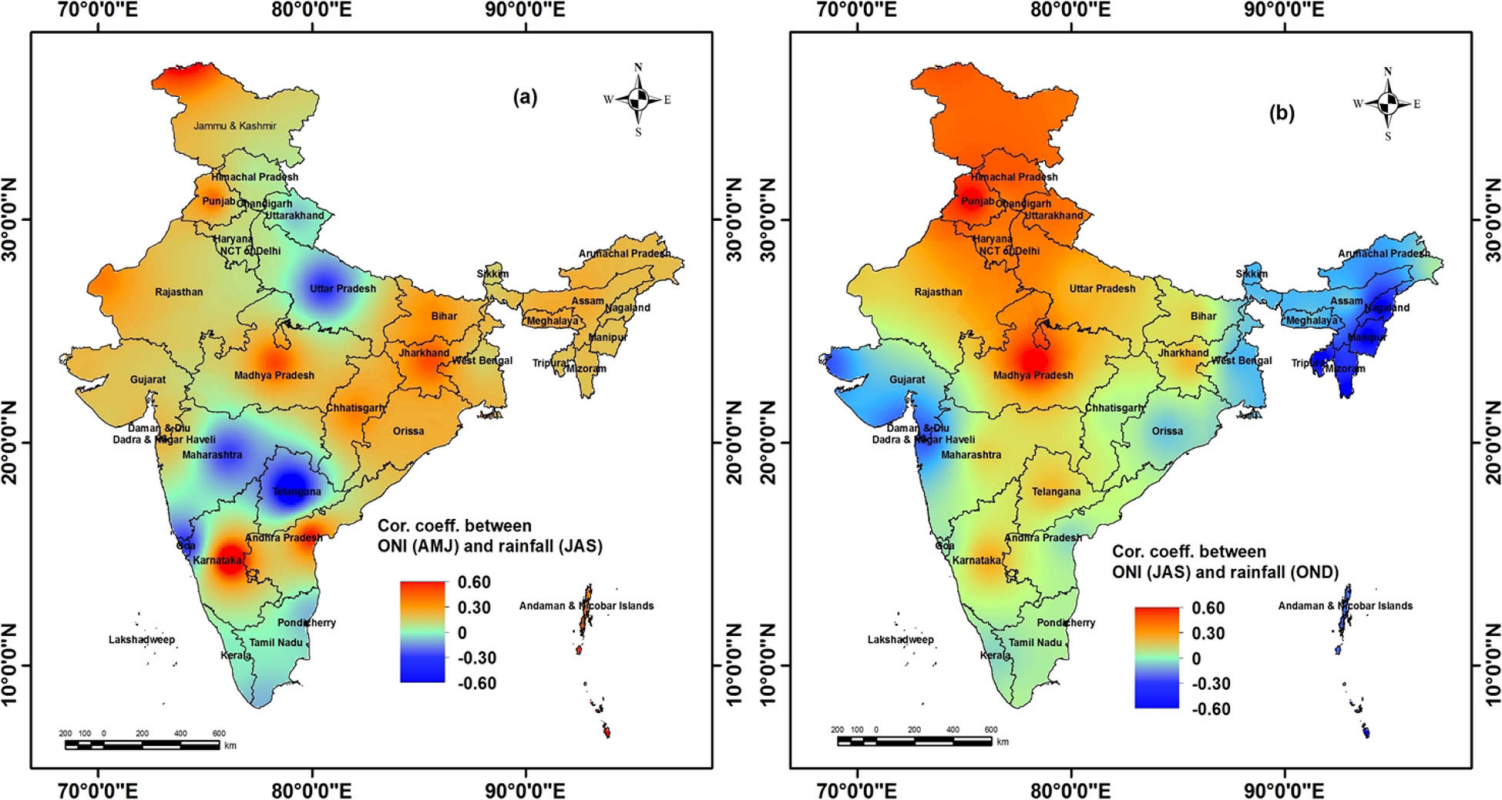
**a** Correlation coefficient between positive summer Oceanic Niño Index (April, May, June) and rainfall index of monsoon (July, August, September) season and (**b**) ONI of July, August, September and rainfall of post monsoon (October, November, December). The shapefiles used for these maps were open access provided by ESRI ArcGIS Online (2018). The maps were generated using ESRI ArcMap 10.2

The relationship between positive summer ONI (April, May, June) and rainfall index of monsoon (July, August, September) and post monsoon (October, November, December) season is shown in Fig. 2. During positive summer ONI, when there was positive El Niño (positive ONI) influence, negative correlation with rainfall (deficit rainfall than normal) was observed in northern Indian plains (Uttar Pradesh, Himachal Pradesh), southern India (Kerala, Tamil Nadu), western part of India (Haryana, Delhi, Chandigarh), and western coastal states (Goa, Maharashtra, Telangana). A negligible relationship was found in the states of Arunachal Pradesh, Maharashtra, eastern Rajasthan, east and west Madhya Pradesh, whereas, the western states (i.e., Rajasthan, Gujarat), northeastern (i.e., Arunachal Pradesh, Assam, Meghalaya) northern (i.e., Jammu and Kashmir, Punjab), and eastern states (i.e., Bihar, Jharkhand, West Bengal) showed positive correlation indicating excess rainfall than normal.

The association between positive monsoon ONI (July, August, September) and rainfall index (October, November, December), was found negatively correlated in the north eastern states (Assam, Nagaland, Meghalaya, Tripura, Manipur), eastern part of India (West Bengal, Orissa), western part (Gujarat), and western coastal states (Maharashtra, Kerala, Goa) indicating deficit rainfall than normal. On the other hand, the northern states (Haryana, Delhi, Chandigarh, Himachal Pradesh, Jammu and Kashmir, Punjab), Madhya Pradesh, Rajasthan and Telangana showed positive correlation indicating excess rainfall than normal.

### Correlation between rainfall index and dengue case index

**Fig. 3:**
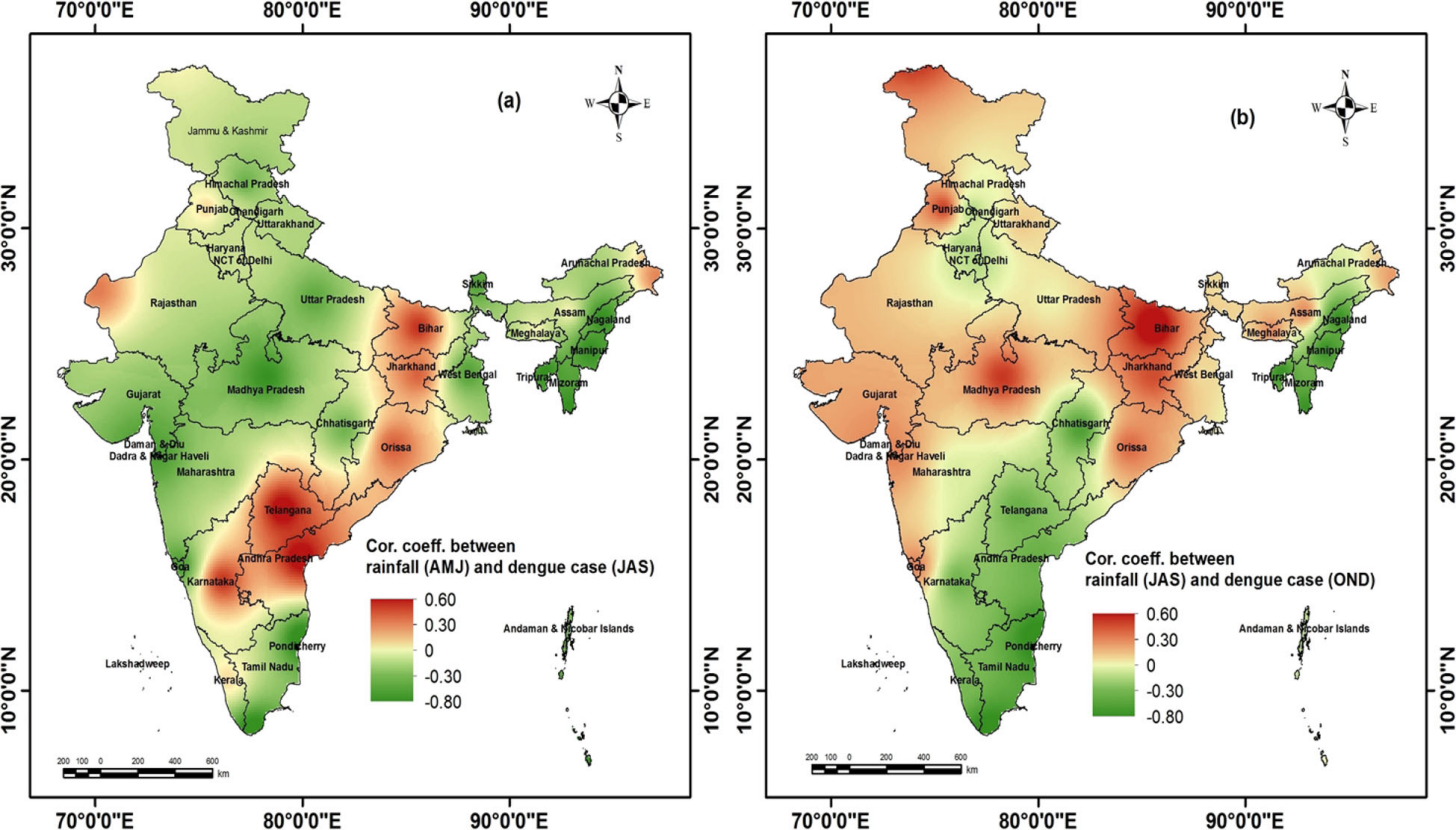
Relationship between (**a**) rainfall index (April, May, June) and dengue case index in monsoon season (July, August, September); (**b**) rainfall index (July, August, September) and dengue case index in post-monsoon season (October, November, December). The shapefiles used for the map were open access provided by ESRI ArcGIS Online (2018). The maps were generated using ESRI ArcMap 10.2

The correlation between rainfall index (July, August, September) and dengue case index (July, August, September) showed negative correlation in most of the northeastern states (Nagaland, Manipur, Mizoram, Tripura), northern states (Haryana, Himachal Pradesh, Chandigarh and Delhi), Chhattisgarh, western part (Gujarat, eastern Rajasthan), and western coastal states (Mahasrhtra, Goa) (Fig. 3) indicating a increase in dengue with rainfall deficit in the monsoon season. However, when the analyses in respect of Delhi was done with lag periods, a lag period of 1–3 month was found between ‘rainfall index’ and ‘dengue case index’. On the other hand in the eastern states (Bihar, Jharkhand), eastern coastal states (Andhra Pradesh, Telangana, Orissa), and western part of Rajasthan, positive correlation was found indicating dengue outbreaks during excess rainfall.

Further, Fig. 3 shows negative correlation between post-monsoon rainfall (October, November, December) and dengue case index (October, November, December), in the northeastern states (Nagaland, Manipur, Mizoram, Tripura), eastern coastal states (Tamil Nadu, Pondicherry, Andhra Pradesh, Talangana), northern India (Haryana, Chandigarh and Delhi), and southern part of western coast (Kerala, Karnataka) indicating increase in dengue cases due to post monsoon rainfall deficit. The eastern states (Bihar, Jharkhand, West Bengal), Madhya Pradesh, northern Chhattisgarh, Orissa), western part of India (i.e., Gujarat, Rajasthan), and western coastal states (western Maharashtra, Goa) register positive correlation between post-monsoon rainfall and dengue case index, representing an intensification in dengue during excess post-monsoon rainfall. However, when the analyses were undertaken with lag period, it was found that with 1–3-month lag period higher correlation was found between rainfall index and dengue case index.

### Correlation between positive ONI and dengue case index

**Fig. 4:**
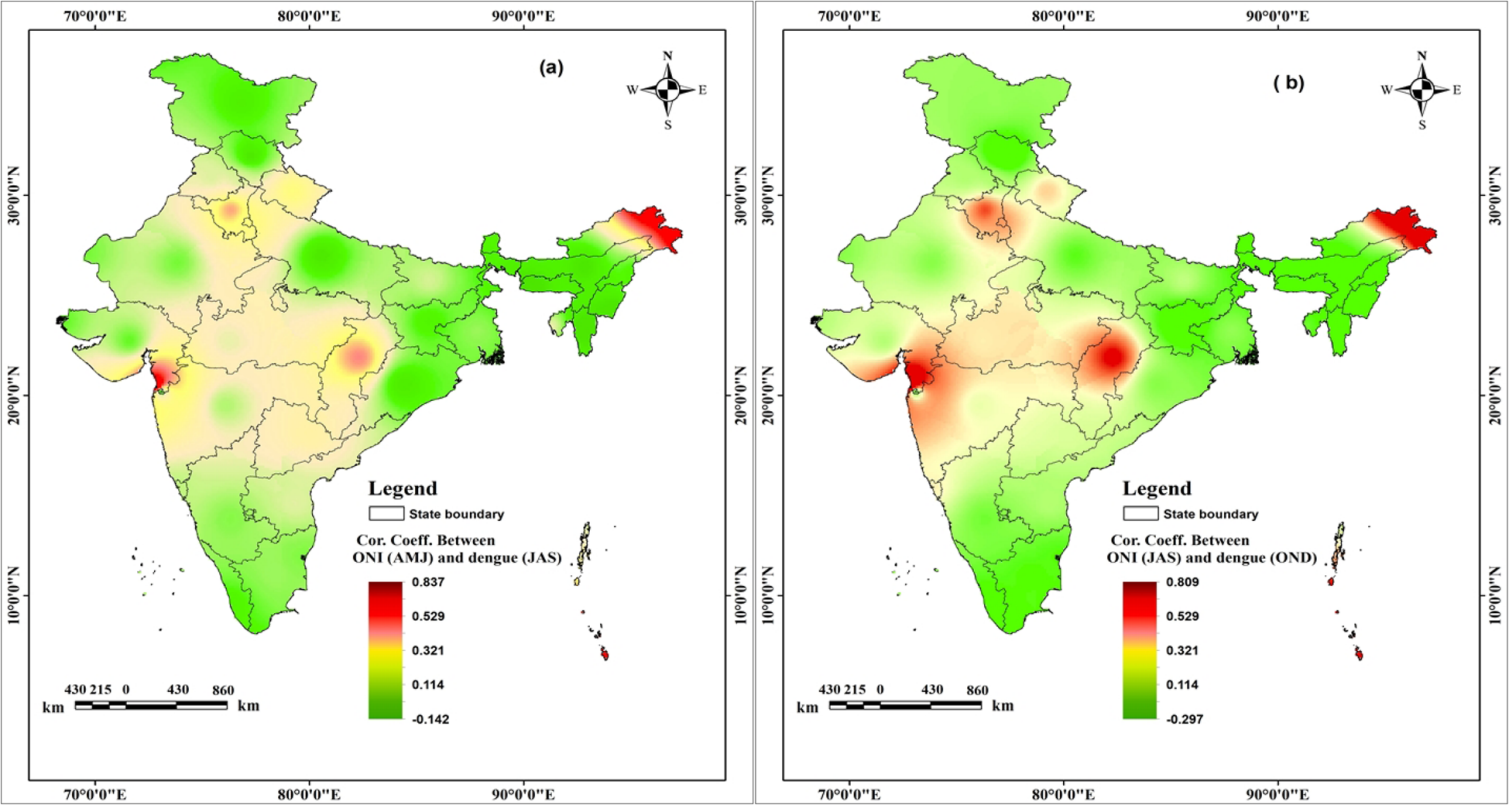
Correlation coefficient between (**a**) positive Summer ONI (April, May, June) and dengue case index in the monsoon season (July, August, September) and (**b**) positive monsoon ONI (July, August, September) and dengue case index in the post monsoon season (October, November, December). The shapefiles used for the map were open access provided by ESRI ArcGIS Online (2018). The maps were generated using ESRI ArcMap 10.2

The relationship between positive Summer ONI (April, May, June) and dengue case index (July, August, September, lag period of 3 months) has been shown in Fig. 4a, indicating that most of the states like Arunachal Pradesh, Chhattisgarh, Haryana, Uttarakhand, Andaman and Nicobar Islands, Delhi, Daman and Diu show positive correlation (0.69 to 0.84). These states are likely to register dengue outbreaks in monsoon season after high positive summer ONI. On the other hand, negative corrleation was found in the states of Assam, Himachal Pradesh, Meghalaya, Manipur, Mizoram, Uttar Pradesh, Orissa, Jammu and Kashmir and Andhra Pradesh indicating decrease in dengue cases after positive summer ONI (strong El Niño). The states like Gujarat, Karnataka, Rajasthan, Dadra, and Nagar Haveli and Puducherry show ‘poor correlation’ indicating negligible impact of summer El Niño on dengue cases (Fig. 4a).

The mapping of correlation coefficient between positive monsoon ONI (July, August, and September) and dengue case index (October, November, and December) is shown in Fig. 4b. A positive correlation (0.40–0.68) was found in the states of Arunachal Pradesh, Chhattisgarh, Haryana, A & N Islands, Daman and Diu, Delhi and Haryana indicating that with positive monsoon ONI increase in dengue cases is likely to be witnessed in post monsoon months i.e. October, November and December. On the other hand, negative correlation was found in the states of Assam, Himachal Pradesh, Andhra Pradesh, Jharkhand, Meghalaya, Manipur, Nagaland, Sikkim and Mizoram reflecting decrease in dengue cases after positive monsoon ONI (monsoon El Niño). Rest of the states show ‘very low or no correlation’.

### Predicted dengue outbreak states based on ENSO outlook of 2020

**Fig. 5:**
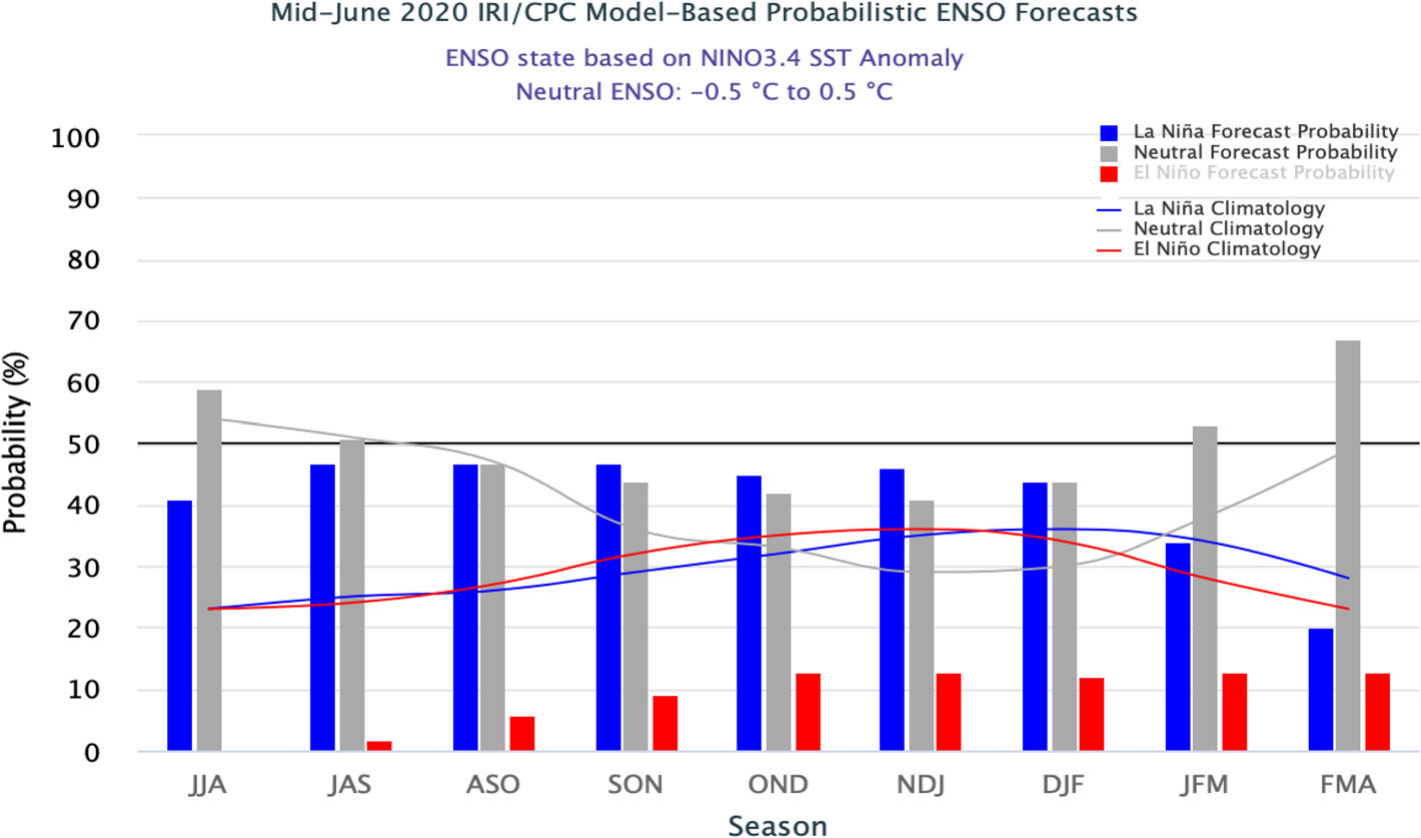
Model based Probabilistic ENSO forecasts (Source: CPC/IRI, mid-June, 2020). Y-axes are showing ENSO probabilities for positive ONI (red colour bar), Neutral (shaded colour block), and negative ONI (blue colour bar) and X-axes represent 3 months’ average block. The graph indicates from July 2020 to March 2021, there is moderate to very high chances of neutral ENSO situation in India (Source: http://www.cpc.ncep.noaa.gov/)

**Fig. 6:**
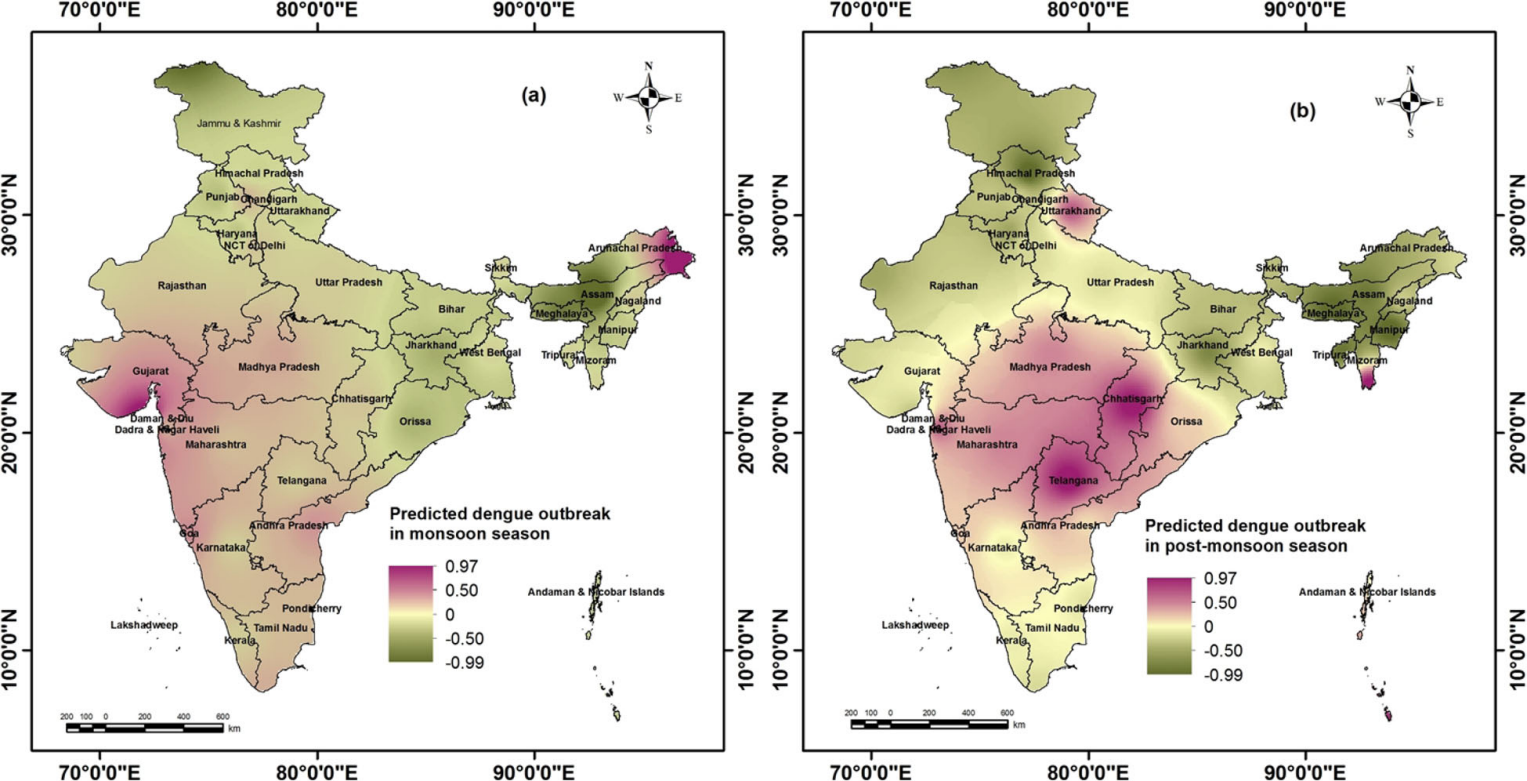
Predicted outbreak of Dengue in (**a**) monsoon season (July, August, September); and (**b**) post monsoon season (October, November, December), 2020 based on positive summer and monsoon ONI values of 2020. The shapefiles used for the map were open access provided by ESRI ArcGIS Online (2018). The maps were generated using ESRI ArcMap 10.2

As per probabilistic ENSO outlook (CPC/IRI) (Fig. 5) extracted in mid-June 2020, there are moderate to high chances (40–58%) of normal condition (neutral) between July 2020 to December 2020 indicating the possibility of normal rainfall during monsoon and post-monsoon season in India. Therefore, the north-eastern state i.e. Arunachal Pradesh, western part (Gujarat, western Madhya Pradesh), eastern coast (Andhra Pradesh, Tamil Nadu), and northern part of western coast (Maharashtra, Daman and Diu, Goa) are likely to have dengue outbreaks during July to September (monsoon season) of 2020. The eastern coastal states (Andhra Pradesh, Telangana, Orissa), central India (Madhya Pradesh, Chhattisgarh), western coast (Goa, Maharashtra, Daman and Diu, Dadra Nagar Haveli), north-eastern states (Mizoram, Tripura), and the state of Uttarakhand are likely to have dengue outbreaks during post-monsoon months from October to December 2020, provided surveillance and interventions remain unchanged. (Fig. 6a and b). Moreover, in the early 2021 i.e. February 2021 to March 2021, very high probability of neutral scenario of ENSO is projected.

### Local-scale analysis results

**Fig. 7:**
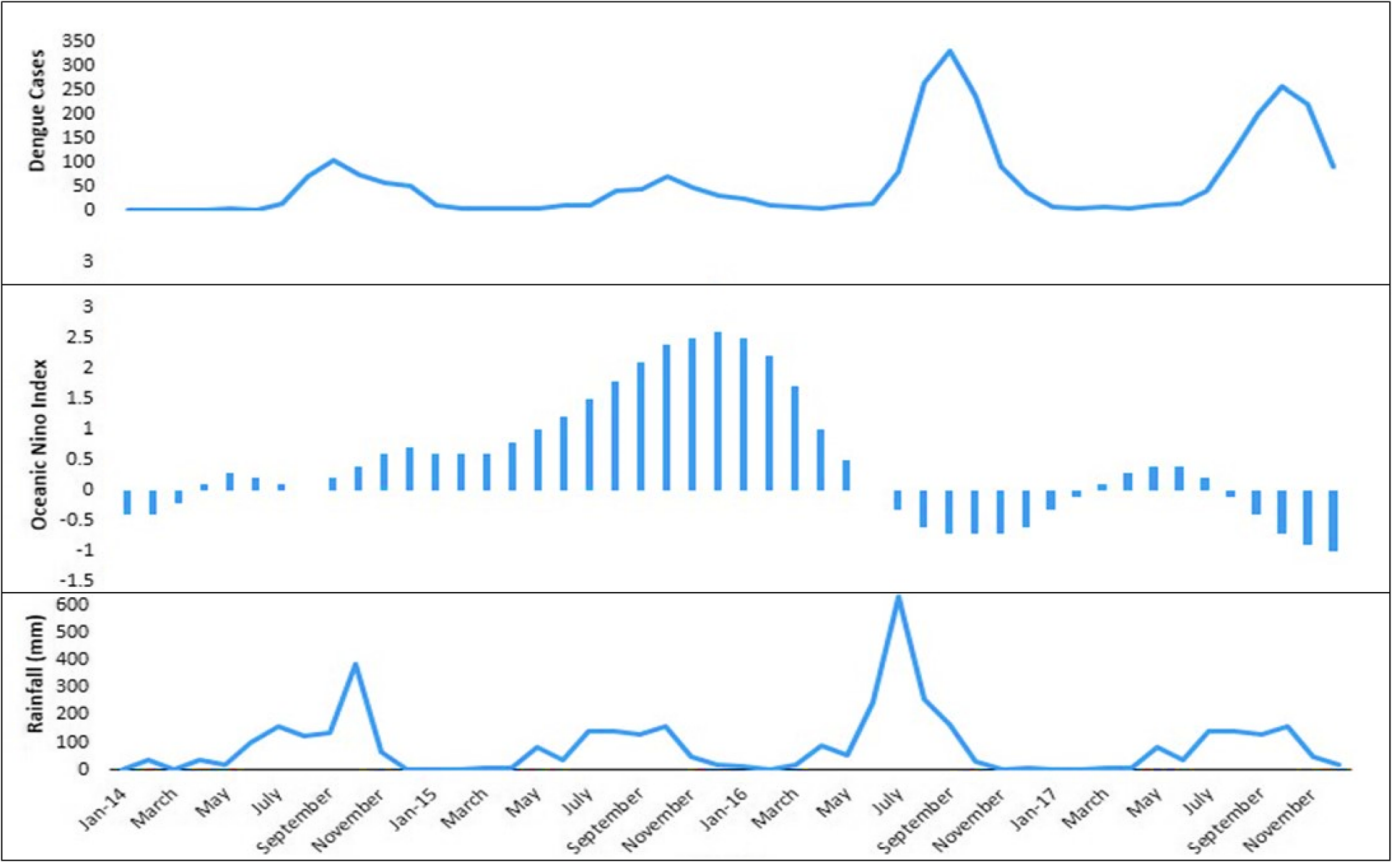
Reported monthly cases of dengue, ONI, and rainfall in Visakhapatnam for the year of 2014–2017

**Fig. 8:**
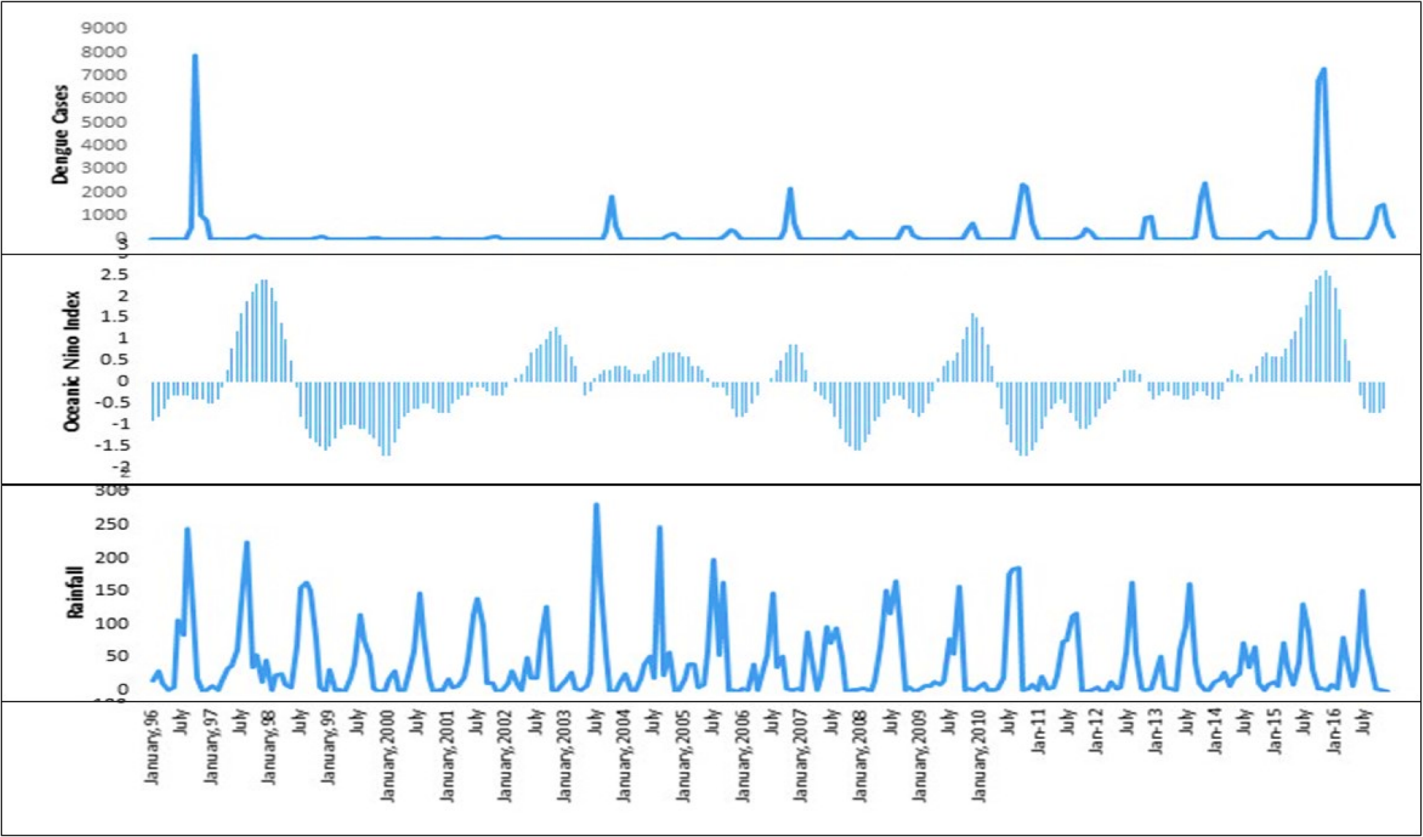
Reported monthly dengue cases, ONI, and rainfall in Delhi, 1996–2016

As per results, some states (Arunachal Pradesh, Chhattisgarh, Haryana, Uttarakhand, Andaman and Nicobar Islands, Delhi, Daman and Diu) are positively correlated with positive ONI while some are negatively correlated (Assam, Himachal Pradesh, Meghalaya, Manipur, Mizoram, Uttar Pradesh, Orissa, Jammu and Kashmir and Andhra Pradesh). Further, local scale analysis, by identifying one district each from positively and negatively correlated states, was done to find out the association between positive ONI and dengue cases. It was found that in Delhi and Visakhapatnam, 1–3 months lag between positive ONI and reported dengue cases was observed. The results also showed that 2-month lag was found between monthly rainfall index and dengue cases in both the places (Delhi and Visakhapatnam cities). Lag month-wise correlation values between dengue cases and positive ONI, rainfall are shown in Table 1. The largest El Niño events (ONI) resulted in very limited number of dengue cases in Visakhapatnam in 2015 (Fig. 7) while Delhi recorded a very high number of dengue cases in 2006, 2009, 2015 (Fig. 8). Thus, the results show that the association between positive ONI and occurrence of dengue cases in identified areas is statistically significant based on hypothesis test.

**Table 1:** Correlation coefficients between monthly positive ONI and Dengue Cases; and rainfall and Dengue Cases (2014–2018) with different lag periods in Delhi and Visakhapatnam (*indicates *p* value at 5% significance level)

Lag-month	Delhi (Correlation coefficient, ‘r’)		Visakhapatnam (Correlation coefficient, ‘r’)	
	Positive ONI & dengue cases	Rainfall & dengue cases	Positive ONI & dengue cases	Rainfall & dengue cases
0	0.368	−0.053	−0.541	0.316
1	0.429*	0.189	−0.545*	0.661
2	0.401*	0.412*	−0.552*	0.742*
3	0.217	0.285	−0.537*	0.501*
4	−0.152	0.093	−0.498	0.141
5	−0.178	0.079	−0.44	−0.111
6	−0.131	0.088	−0.431	−0.293
S Error	0.105	0.058	0.019	0.147

## Discussion

A significant relationship was found between ENSO and inter-seasonal and inter-annual variability of dengue via significant negative deviation of rainfall and drier conditions suggesting that El Niño, La-Nina conditions significantly influence monsoon and post-monsoon rainfall and subsequently dengue incidence in India. Moreover, the state-wise analysis of dengue reveals that upsurge in dengue cases was observed during 2003, 2005, 2010, and 2016 El Niño in India. Our study shows that in India there is a very high possibility of dengue outbreaks in the north-eastern states, western part, western coast, and northern part of western coastal states during the monsoon season, and in eastern coasts, central India, western coast, and north-eastern states during the post monsoon season. The outbreaks in this region are likely to be due to rainfall and the availability of humidity in the environment directly influences the availability of breeding habitats for the existence of mosquitoes during this season [21]. Due to great geographical variations in India, transmission of dengue in all the states is not equally influenced by the seasonal rainfall (excess or deficit). In one region, deficit rainfall causes dengue outbreaks while in other very high rainfall may lead to outbreaks.

A number of studies undertaken in South Asia [14, 16, 18, 25] and Oceania, South America, and in Central America [15, 26, 27] revealed the possibility of early warning of dengue outbreaks based on ENSO phases. The studies have affirmed that ENSO phases and intra-seasonal and inter-annual climate prediction might offer preparedness and response for early management and control of outbreaks of dengue [28].

Previous studies have established close relationship between ENSO phases and fluctuation of seasonal rainfall in India [11, 29], therefore, understanding of rainfall variability could explain the regional variation and complexity of dengue outbreaks in India. During El-Niño phase, the warmer temperature is likely to result in storage of water which leads to breeding of Aedes vector as well as fasten the extrinsic incubation period resulting in outbreaks, replication rate of dengue viruses and by lengthening the lifespan of mosquitoes [30]. Nevertheless, during El-Niño phase, temperature anomalies affect dengue incidence negligibly without the contribution of rainfall variability. However, with 3 months lag after Summer ONI, strong rainfall variabilities affect dengue incidence by the changes in the suitability of mosquito breeding sites and survival of vector species [31]. In addition, in some areas, collection and storage rainwater due to the poor garbage disposal and unavailability of piped water affects the suitability of mosquito habitats and subsequent mosquito transmission. Prolonged drier conditions during El-Niño years, water supply problems and increasing storage of water gradually increase the suitability of breeding habitats of vectors [32, 33].

The Indian Ocean Dipole (IOD), (known as the Indian Niño), is an irregular oscillation of SST in which the western Indian Ocean becomes alternately warmer (positive ONI) and then colder (negative ONI) than the eastern part of the ocean [34]. The IOD events have been significantly influencing the rainfall during summer monsoon season in India [34], and the values of IOD index are closely associated with ONI [35], therefore, only ONI was considered in the present study. Moreover, other factors like local climatic variables and ‘epochal variation of monsoon rainfall’, are also strongly correlated with Indian seasonal rainfall [36–38]. The possibility of forecast of outbreak of dengue based on state level analysis is a limitation, however, the phase-wise probability of predicted dengue outbreaks map of the present study should possibly guide primary input of dengue outbreaks and local-scale analysis.

A local-scale (city/district level) studies with month-wise association between IOD and local weather variables, are warranted where the low or negligible correlation between dengue cases and ENSO events has been found. Moreover, the weekly lag analysis may improve the results than monthly lag analysis undertaken in the present study. It is also proposed that with the help of ENSO based analysis at district level, early warning of outbreaks of dengue should be possible in India.

## Conclusions

The current research has shown that the dengue outbreaks are highly correlated with ENSO, monsoon and post-monsoon rainfall particularly in the states like Arunachal Pradesh, Chhattisgarh, Haryana, Uttarakhand, Andaman and Nicobar Islands, Delhi, Daman and Diu. While the states like Assam, Himachal Pradesh, Andhra Pradesh, Meghalaya, Manipur, Mizoram, Uttar Pradesh, Orissa, Jammu and Kashmir shows a negative correlation between summer El Niño and dengue incidence. The phase-wise probability of predicted dengue outbreaks maps generated in the present study should possibly guide the national programme in pre-empting the impending outbreaks of dengue at coarse level. Further, improvement of the results by employing local-scale analysis across India could help in the early warning system of dengue outbreaks via timely response and early preparedness by the national programme. For better understanding of the association between ENSO and dengue, a comprehensive month-wise association with IOD and rainfall at microscale (district/city-level) is needed.

## Data Availability

The datasets used and/or analyzed in the current study are openly available for public access. The sources and links are given in methods section and also in reference no 22–24. In respect of local scale analysis of cities i.e. Delhi and Vishakhapatnam, the procured monthly data of dengue is available with corresponding author and can be provided on reasonable request.

## Abbreviations

ENSO: El Niño Southern Oscillation
ONI: Oceanic Niño Index
IOD: Indian Ocean Dipole
NVBDCP: National Vector Borne Disease Control Programme
